# Promoting student voice by involving vocational education students and staff in citizenship education development: a participatory action research study

**DOI:** 10.1007/s10833-024-09523-y

**Published:** 2025-01-24

**Authors:** Esther M. A. Geurts, Rianne P. Reijs, Hélène H. M. Leenders, Maria W. J. Jansen, Christian J. P. A. Hoebe

**Affiliations:** 1 Department of Social Medicine, Care and Public Health Research Institute (CAPHRI), Maastricht University, Maastricht, The Netherlands; 2 Department of Youth Health Care, Living Lab Public Health Mosa, South Limburg Public Health Service, Heerlen, The Netherlands; 3 Department of Pedagogical Studies, Fontys University of Applied Sciences, Sittard, The Netherlands; 4 Youth Education for Society, Centre of Expertise, Fontys University of Applied Sciences, Sittard, The Netherlands; 5 Department of Health Services Research, Care and Public Health Research Institute (CAPHRI), Maastricht University, Maastricht, The Netherlands; 6 Living Lab Public Health Mosa, South Limburg Public Health Service, Heerlen, The Netherlands; 7 Department of Sexual Health, Infectious Diseases and Environmental Health, Living Lab Public Health Mosa, South Limburg Public Health Service, Heerlen, The Netherlands; 8 Maastricht University, PO Box 616, Maastricht, 6200 MD The Netherlands

**Keywords:** Curriculum negotiation, Participatory action research, Student participation, Student voice, Vocational education and training

## Abstract

Introduction: Despite decades of school improvement efforts, maintaining lasting change in schools remains challenging. So far, traditional interventions have been unsuccessful in recognising schools’ unique and complex contexts, which is why a shift towards a more reciprocal, emergent, and contextualised approach is necessary. Objective: Our aim is to develop citizenship education with students and staff in vocational education and training (VET), which increases student voice and fits the complex school system. Methods: We involved students and staff in citizenship education development and identified relevant factors that influenced this process. This participatory action research (PAR) study used a retrospective design assessing logbook entries, observations, interviews, and focus group discussions collected between September 2020 and September 2023 in four study programmes from one VET institution in the Netherlands. Thematic analysis was used to code the datasources. Results: Collaboration between students and staff flourished while using the curriculum negotiation tool. Relevant factors influencing the process were: alignment between project and participants’ priorities and goals, receptivity to student and teacher voices, leadership engagement, and tensions and intentions about roles and responsibilities between participants. Conclusions: This study emphasises the promise of involving students and staff in educational development. When teachers startedworking collaboratively with students, many of their initial doubts decreased, which led to renewed motivation for becoming student voice advocates. PAR has the potential of starkly disrupting the existing status quo and breaking through ingrained patterns within complex systems to ensure influence for students from all levels of education.

## Introduction

Despite decades of school improvement efforts and endless cycles of reform (Trombly, 2014), maintaining lasting change in schools remains exceedingly challenging (Hargreaves & Fink, 2012). So far, traditional interventions have been unsuccessful in recognising schools’ unique and complex contexts. Indeed, previous research (Askell-Williams, 2017; Askell-Williams & Murray-Harvey, 2016) underlined that school improvement interventions were generally offered as “externally-designed, relatively stand-alone programmes that did not fully account for the nature of schools” (Koh & Askell-Williams, 2021, p. 282). Yet, as interventions and the contexts in which they are implemented become increasingly complex (Waterlander et al., 2020), a shift towards a more reciprocal, emergent, and contextualised approach is necessary, in which intervention development is an ongoing and interactive process rather than a goal in itself (Mason, 2008; McQuillan, 2008). In order to achieve that, schools should be conceptualised as complex adaptive systems and by extension as collections of individual agents with freedom to act in ways that are not always predictable and whose actions are interconnected (Plsek & Greenhalgh, 2001). As both the system and its agents are capable of continuous adaptation and change, their behaviour may differ over time (Signal et al., 2013; Waterlander et al., 2020) leading to the emergence of new and sometimes unexpected behaviours (Mason, 2008). Complex adaptive systems are further characterised by treating tension and paradox as natural phenomena in such a way that the system and its agents frequently alter their goals and priorities, sometimes resulting in seemingly contradictory needs (Plsek & Greenhalgh, 2001). In keeping with the notion of complex adaptive systems, there is increasing acknowledgment of the importance of involving local stakeholders in co-creation and decision-making concerning the development and implementation of interventions. Such interventions are more likely to align with local understandings, context, and practices, and in turn, are believed to hold more promise in terms of effectiveness, relevance, and acceptability (Gregg et al., 2008; Izumi et al., 2013; O’Mara-Eves et al., 2015).

Participatory approaches have been successful in guiding school improvement intervention development (e.g., Berg et al., 2018; Coll et al., 2018; Fielding, 2001). One such approach is Participatory Action Research (PAR), which collaborates with stakeholders in studying issues that are relevant to them and consequently enhances their abilities to take action within their own communities (Cammarota & Fine, 2010). PAR integrates the expertise of academic professionals with the strengths and lived experiences of stakeholders (Gosin et al., 2003). Mutual and joint learning cycles are created in which participants continuously implement, observe, reflect, plan, adjust, and act in order to improve practice. In school settings, this means that students, staff, and researchers engage in producing and integrating knowledge, while coming up with diverse insights to foster school improvement, which are subsequently tested in real-life settings. In accordance with the nature of complex adaptive systems, PAR is characterised by an emergent design and is thought to be an iterative process (Lang et al., 2012; Regeer & Bunders, 2009; Rodríguez & Brown, 2009).

PAR involves actively privileging student voice in school improvement efforts (Caraballo et al., 2017; Fine et al., 2007) and, as such, emphasises: “that young people have unique perspectives on learning, teaching and schooling; that their insights warrant not only the attention but also the responses of adults; and that they should be afforded opportunities to actively shape their education” (Cook-Sather, 2006). Previous studies demonstrated that when students actively participated in the teaching and learning process, it contributed to greater involvement, commitment, engagement, and enthusiasm. Such approaches also fostered a sense of joy and investment, which, in turn, boosted students’ confidence, sense of ownership, and empowerment (Enright & O’Sullivan, 2010, 2012; Guadalupe & Curtner-Smith, 2020; Howley & Tannehill, 2014; Pennisi, 2013; Shilcutt et al., 2021, 2022; Wahl-Alexander et al., 2016). Student voice in the context of teaching and learning involves including students in co-creating and making decisions about tasks usually reserved for teachers, such as planning the learning process, assessment, and curriculum making (Müller-Kuhn et al., 2021). However, students have rarely been allowed to share their views, let alone participate in decision-making, especially in teaching and learning (Geurts et al., 2024). Moreover, when student voice is considered in teaching and learning, it is usually solely incorporated in initial or final activities, or after major decisions have already been made (Dyson, 1995; Oliver & Kirk, 2015). Being involved in co-creation and decision-making is believed to be especially worthwhile for vulnerable students (Brooker & MacDonald, 1999; Cook-Sather, 2002). Nevertheless, most existing studies concerning adolescents and young adults exclusively target university students (e.g., Bergmark & Westman, 2016; Bovill, 2014; Carey, 2013). One group which is likely to have vulnerabilities but has consistently been overlooked in student voice research is students in vocational education and training (VET; Geurts et al., 2024). VET institutions play an important role in providing citizenship education, which helps students acquire the knowledge, skills and attitudes needed to engage actively and responsibly in a democratic society. Citizenship education provided the perfect circumstances for introducing a student voice project as both strive for student empowerment. Our aim is to develop citizenship education together with VET students and staff which increases student voice and fits the complex school system. Although collaboratively developed school interventions are becoming more popular, there is a lack of guidance on how to meaningfully involve students and staff in such efforts within complex systems (Moore et al., 2019). Therefore, our research questions are: (1) How to involve students and staff in co-creation and decision-making regarding citizenship education development in VET? and (2) What are relevant factors that influence the process of involving students and staff in co-creation and decision-making concerning citizenship education development in VET?

## Methods

### Study design

This qualitative study used an explorative and retrospective design based on thematic analyses of logbook entries, observations, interviews, and focus group discussions collected between September 2020 and September 2023 in four study programmes from one large VET institution with multiple locations in the south of the Netherlands. Initially, the final product was not predefined and it was up to the stakeholders to design and develop an innovation which would boost the citizenship education curriculum, increase student voice in teaching and learning, and fit the unique context of each study programme. The principal researcher (EG) closely participated in the facilitation and implementation of involving students and staff in citizenship education throughout the project, which consisted of three phases. During phase 1, staff explored and identified the focus during monthly workgroup sessions, while implementing action activities in order to involve students. Phase 2 focused on action planning and design and resulted in a draft of the curriculum negotiation tool, which allowed students to design, develop, and deliver citizenship education lessons for their peers. The tool was piloted, implemented, and evaluated during phase 3 (for more information, see Geurts et al., 2023). This study generally focuses on the first two phases and thus on the process of involving students and staff from various study programmes in citizenship education development. As previously mentioned, Participatory Action Research was the methodological framework for this study.

All participants provided written informed consent. To safeguard their privacy, all records were anonymised and data which may reveal their identities were deleted from the transcripts. The study’s research proposal was reviewed by the Medical Ethical committee of FHML-REC/2021/075, which determined that formal ethical approval was not necessary.

### Educational setting and participants

VET in the Netherlands is offered at four different levels. Level 1 consists of assistant training and lasts up to one year. Level 2 provides basic vocational training and lasts two to three years. Level 3 is concerned with intermediate vocational training and lasts three to four years. Level 4 is the highest attainable level in VET and focuses on management or specialist training lasting up to four years. This level also provides admission to higher education (EP-Nuffic, 2015). Pre-vocational education students must at minimum complete a VET programme at level 2 in order to obtain basic qualification before entering the labour market. Previous research usually focused on the intermediate and advanced levels (i.e., level 3 and level 4) or treated VET students as one homogeneous group. This project specifically targeted VET students of the basic level (i.e., level 2), because these students are believed to be extra vulnerable and have formerly been overlooked. Level 1 was not included due to the short window of opportunity (i.e., these programmes only last one year), a high degree of student turnover (i.e., students come and go throughout the schoolyear), and limited interest from the participating institution.

In addition to preparing students for a profession, Dutch VET institutions are legally obliged to instil necessary knowledge, skills, and attitudes, which, in turn, allows these young adults to participate in a democratic society as active and responsible citizens. This duty takes shape and becomes concrete during citizenship education.

The participating VET institution caters to approximately 17,000 students divided among 230 study programmes in ten locations; 32 of these study programmes are at level 2. The institution is organised in a decentralised manner, meaning that study programmes under the leadership of educational managers have a great deal of power in making decisions, arranging finances, determining priorities, and establishing partnerships with external partners. Educational leaders are next in line and are responsible for educational quality assurance and professional development of staff. This study included students, teachers, managers, and educational leaders from four level 2 study programmes: Sports and Recreation (± 125 students), Hairdressing (± 140 students), Home and Personal Care Assistance (± 350 students), and Food and Hospitality (± 115 students). These study programmes were selected after their managers had expressed an interest in joining the project.

Five classes eventually participated in the implementation of the curriculum negotiation tool: two classes from Home and Personal Care Assistance (students mainly from inclusive education, special needs education, and/or entry level), two classes from Hairdressing (one class of Syrian refugees and one class of ‘regular’ Dutch-speaking students) and one class from Sports and Recreation (students mainly from inclusive education, special needs education, and/or entry level).

### Data collection

Data were collected using various data sources: logbook entries, observations, semi-structured interviews with teachers, and focus groups discussions with students. First, the number and essence of all contacts were tracked and documented in logbook entries (i.e., one per study programme, so four logbooks in total). Second, observations were collected during lessons, staff meetings, and on-site visits. These included fieldnotes and reflections. In total, 95 lessons and 81 meetings with staff were observed. Third, eight semi-structured interviews were conducted with every teacher (i.e., eight interviews with six teachers; the two teachers who participated in the first implementation round were interviewed twice: once after the planning sessions and once after the student-led sessions), who had participated in the implementation of the curriculum negotiation tool. These interviews focused on personal beliefs, goals, feelings, successes, and challenges in working with the tool. Moreover, teachers reflected on its impact on their students, their own role, and takeaways. Every interview was recorded and transcribed verbatim. Fourth, during eight focus group discussions as part of the final lesson of the student-led curriculum, 45 students (i.e., five to ten per group) shared their views on working with the curriculum negotiation tool. During the first implementation round, every focus group discussion was recorded and transcribed verbatim (i.e., three in total). However, technical issues with recording multiple individuals in a classroom or occasionally outside or in a sports or care facility severely complicated the recording and transcription process. That is why in the ensuing rounds, only notes were taken during the remaining five focus group discussions, which had the benefit of providing students with an extra sense of safety and confidentiality, encouraging them to speak more freely and openly. See Table 1 for an overview of the collected data per study programme and per phase.

**Table 1:** Overview of all collected data per study programme and per phase

	Sports and recreation	Hairdressing	Home and personal care assistance	Food and hospitality
**PHASE 1: EXPLORING AND IDENTIFYING THE FOCUS (2020/2021)**				
*# of observed lessons*	13	2	14	3
*# of workgroup sessions*	6	5	4	5
*# of extra meetings with staff*	3	7	7	5
*Institution wide meetings*	8			
*Action activities*	Teachers consulted students regarding topics to discuss in class.	–	–	Study afternoon in which teachers and students came up with topics for future citizenship education days.
**PHASE 2: ACTION PLANNING AND DESIGN (2021/2022)**				
*# of observed lessons*	–	2	4	–
*# of meetings with staff*	3	2	4	7
*Institution wide meetings*	4			
*Action activities*	Teachers consulted students regarding topics to discuss in class.	–	–	Three citizenship education days
**PHASE 3: PILOT**,** IMPLEMENTATION AND EVALUATION (2022/2023)**				
*# of meetings with staff*	5	3	–	3
*Action activities*	Used curriculum negotiation tool	Used curriculum negotiation tool	Used curriculum negotiation tool	During a citizenship education day, the researcher and educational manager engaged 6 classes in coming up with topics for future citizenship education days.
*Curriculum negotiation tool (Geurts et al.*, 2023*)*				
*# of classes that used tool*	2	2	1	–
*# of planning sessions*	9	13	8	
*# of student-led lessons*	8	11	8	
*Total # of sessions using tool*	17	24	16	
*# of interviews with teachers*	2	3	3	
*# of focus groups with students*	2	3	3	

Data triangulation was applied in various ways. Multiple sources were included by combining perspectives from various stakeholders. Moreover, diverse types of data (i.e., observational data, transcripts, and reflections) were collected across time (i.e., by studying four study programmes for three schoolyears) and in various settings (i.e., different locations and group compositions). Additionally, EG engaged in reflexive analysis by reflecting on potential biases, assumptions, and influence on the data collection process.

### Data analysis

We applied thematic analysis (Braun & Clarke, 2006) to code our longitudinal data sources, using the Consolidated Framework for Implementation Research (CFIR) by Damschroder and colleagues (2022) as a guiding framework for the coding process. CFIR provides a comprehensive, structured, and holistic approach to studying how innovations are implemented in real-world and complex settings; it consists of five domains: innovation characteristics (i.e., attributes of the innovation being implemented), inner setting (i.e., organisational culture, climate and readiness for innovation), outer setting (i.e., external factors that influence implementation), characteristics of individuals (i.e., attitudes, motivations and roles of stakeholders), and process of implementation (i.e., steps and activities needed to implement the innovation). CFIR aligns with our objective to develop citizenship education with VET students and staff to increase student voice, as the framework promotes the active involvement of stakeholders throughout the implementation process. Moreover, it is designed to assess factors at multiple levels (i.e., student/staff, study programme and VET institution) and explores how these levels interact and affect the innovation’s success. In this study, the outer setting domain was excluded, because it was beyond our scope. We also decided not to focus on the innovation domain, because as previously mentioned, at the start of the project there was no innovation yet, as it still needed to be developed by the stakeholders. As such, the implementation domain concentrates on the process of involving various stakeholders in citizenship education development, which eventually resulted in the curriculum negotiation tool.

Using Atlas.ti (version 22), the data were coded in chronological order while diverse sources alternated. The thematic analysis (Braun & Clarke, 2006) started with familiarisation of the data, meaning that EG read all collected data (i.e., 222 separate documents) and made a rough division between what was of interest and what was not based on the three CFIR domains. Next, EG used the constructs of the CFIR domains to code the remaining 116 documents. Subsequently, EG reviewed all the data per construct. This round focused on making sure the data were coded correctly, dividing the constructs in subcodes and looking for connections between constructs and/or subcodes. During the final phase, overarching themes were named and defined. We decided to formulate content-driven themes rather than continuing to structure the themes according to the domains, because of enhanced specificity and relevance, improved clarity and increased flexibility in interpretation. Nevertheless, each overarching theme can be linked to one of the CFIR domains.

## Results

First, a general overview of the project and its phases will be provided to gain a deeper understanding of the ways in which students and staff participated in citizenship education development. The development processes, the stakeholders involved, and goals and priorities not only varied considerably between study programmes, but have also changed over time. In order to highlight this diversity and accurately reflect the unique context of each study programme, the relevant factors have been divided in four overarching themes: alignment between project and participants’ priorities and goals (i.e., inner setting domain), receptivity to student and teacher voices (i.e., inner setting domain), leadership engagement (i.e., implementation process domain), and tensions and intentions about roles and responsibilities between participants (i.e., characteristics of individuals domain). Each theme is explored from multiple levels as well as how these levels interact. Due to the varying presence of the themes between programmes as well as over time, each theme is framed neutral. Increased presence of the first three themes improved facilitation of the process. This does not apply to the final theme about tensions and intentions, because these are seen as inherent to complex systems. Within each theme, the most telling subtopics will be discussed and illustrated using various programme-specific and time-specific examples.

### Involving students and staff in citizenship education development

**Fig. 1:**
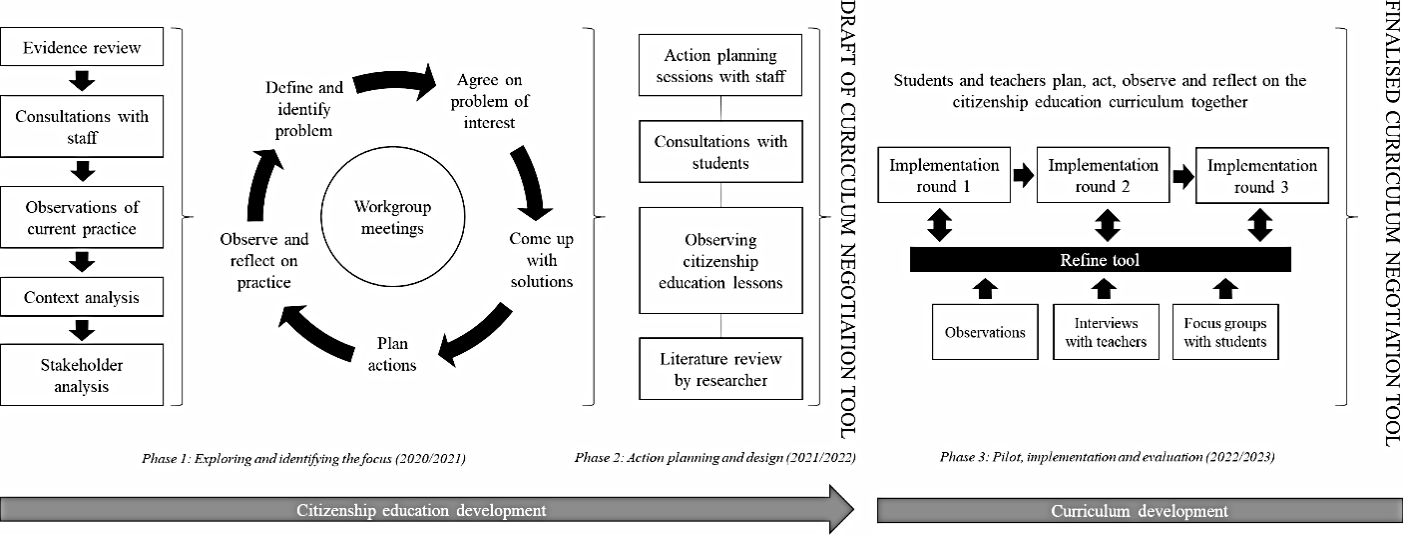
Overview of activities per project phase

Figure 1 provides an overview of the project activities per phase. Phase 1 consisted of observing lessons, getting to know staff, students, and each study programme, performing a stakeholder and context analysis, and setting up a workgroup for each programme consisting of the educational leader, 2–5 citizenship education teachers, and EG. During monthly workgroup sessions, members defined and identified specific problems that they would like to address and subsequently engaged in agenda setting. They also reflected on how students had been involved in teaching and learning so far and how they could include students in the citizenship education development process. During this phase, each study programme enjoyed large degrees of freedom in deciding what kind of intervention to work towards and to what extent students would be involved in its development. While EG took the lead, staff made the final decisions, ensuring that all stakeholders worked collaboratively as partners. Phase 2 proceeded with difficulty as participating staff had a wide range of other pressing tasks and priorities, which impeded meaningful inclusion of students in citizenship education development. As a result, EG became more directive in prescribing minimum efforts in terms of student voice for each programme. Accordingly, teachers started involving their students as advisors during citizenship education lessons. Based on consultations with students and teachers, observed citizenship education lessons, and an extensive literature review (Geurts et al., 2024), EG developed a draft of the curriculum negotiation tool. In Phase 3, the tool was implemented by five teachers from three study programmes (for more information, see Geurts et al., 2023). These teachers were not only implementors, but also became reviewers and partners in continuously improving the curriculum negotiation tool. The tool aided teachers in guiding, supporting and encouraging their students to become the driving force of their own curriculum. Moreover, students were included as reviewers of the citizenship education curriculum, the curriculum negotiation tool, and the developed materials. In conclusion, meaningful student voice primarily transpired while teachers used the tool in the classroom.

### Theme 1: Alignment between project and participants’ priorities and goals

Before the start of the project, the recently installed board of directors had labelled student voice and improving citizenship education as priorities. Pursuant to these ambitions, top management worked collaboratively in forming shared goals to improve citizenship education while involving level 2 students and staff. According to the four managers who eventually signed up for our project, the timing was perfect as the project’s objectives fitted other desired, necessary, or ongoing changes within their programmes. For Hairdressing, for example, it was important that every teacher would be able to provide citizenship education classes since this would make scheduling lessons easier. As such, the project could be used to introduce inexperienced teachers to the subject and make them acquainted with its content. Due to the recent merge of two institutions into one, Home and Personal Care Assistance described their main assignment as generating “one curriculum and one exam plan in which one team across two locations are doing almost the same things.” Joining the project could potentially contribute to this extensive task as well as alleviate some of the organisational burden. Food and Hospitality had a long-standing desire to abandon citizenship lessons and transition to organising citizenship days. Although the programmes’ personal goals do not necessarily have to hinder those of the project, an inherent tension unfolded over the course of the project between promoting student voice in citizenship education development, which was the project’s prime goal, and wanting to achieve alternative objectives, which would directly benefit management. Educational leaders were assigned with getting their teachers on board, which not only turned out to be complicated due to juggling several objectives at once, but also because teachers had hardly been consulted about their needs, let alone whether they wanted to participate in this project at all. Differences in perspectives and needs were not limited to management and teachers, but also occurred between teachers of the same programme. Some teachers wished for flexibility and concrete action, while others demanded peace and clarity. Even though each workgroup spent a great deal of the first year on creating mission alignment as well as shared goals, they did not succeed in overseeing and keeping track of these diverse goals, nor did they find means for connecting them. The fact that those who would develop and implement citizenship education development had not been involved in outlining the original ambitions proved to be too big of a hurdle and often led to confusion, resistance, and/or lack of motivation among participants. Sports and Recreation was an exception in the sense that this study programme had already been reforming their curriculum in order to increase the role of students before the start of the project, which meant that their personal objectives corresponded much better with the overall ambitions.

### Theme 2: Receptivity to student and teacher voices

Even though student voice was the project’s main focus, it quickly became apparent that the desire to increase student voice was not self-evident among the participants. Not everyone immediately grasped the added value of involving students as one teacher from Food and Hospitality tellingly remarked: “The teachers determine what students must do, because we know what to prepare them for.” Another frequently heard sentiment was that teachers were already able to sufficiently relate to their students: “I can empathise fairly well with my students and know what appeals or does not appeal to them, but unfortunately certain topics cannot be avoided” (teacher, Hairdressing). In reality, hardly any requirements are in place regarding specific learning objectives or mandatory content for citizenship education lessons. Nevertheless, many teachers, particularly those from Hairdressing, often mentioned needing to adhere to national rules and regulations. Similarly, their educational leader explained: “A book [for citizenship education] was chosen to cover all topics, to always be sure that we are safe […] The accountability is there […] We do not have to worry about it.” The perceived imminent threat of repercussions in case of any deviations contributed to a culture that was dismissive of student and teacher input. Be that as it may, most participants, from all programmes, expressed an interest in involving students in citizenship education, as the content and activities would be better suited to students’ experiences and needs, which, in turn, would contribute to more self-efficacy, better grades, and increased motivation, involvement, and enthusiasm. Teachers mainly saw opportunities for involving students by discussing topics of interest, potential destinations for site visits, and in-class activities. Some teachers also suggested that students could be involved in designing their own lessons. Nonetheless, most propositions were limited to students sharing their views and opinions. Moreover, when discussing who should be involved, both management and teachers often articulated preferring to involve level 3 and level 4 students, rather than the proposed level 2 students. According to a teacher from Sports and Recreation “we should ask ourselves whether the target population of level 2 students is suitable for student voice activities.” A commonly expressed reason for this was that level 2 students need as well as favour clear structures, because they “are not able to oversee the bigger picture” (teacher, Home and Personal Care Assistance). Moreover, many teachers assumed that level 2 students would not be interested in being involved, nor in co-creating citizenship education. These beliefs were shared at such an extent that participating staff often expressed that, had they been given the opportunity to do the same project with level 3 and level 4 students, the quality, meaning and insights of the input would be greater. Initially, promoting student voice was not a goal in itself, as we had wrongly assumed that our participants would have similar priorities. However, involving students, particularly those from level 2, became one of the leading goals within the project. In conclusion, only Sports and Recreation initially displayed an environment which valued student voice and exhibited openness to change in their eagerness to involve students as more than information providers. Aspects of culture in the remaining study programmes, in contrast, hampered student and teacher perspectives in citizenship education development and contributed to a perception that these programmes were only open to the perspectives of higher level students. Nevertheless, in the end, every study programme except for Food and Hospitality agreed to implement the curriculum negotiation tool in at least one class, which adjusted some of these misconceptions and low expectations as teachers gained first-hand experience in implementing student voice and, in turn, witnessed their students enjoy and purposefully engage in curriculum negotiation.

### Theme 3: Leadership engagement

From the start, educational leaders from Sports and Recreation and from Food and Hospitality showed a constant readiness for change and were involved in implementing day-to-day activities. They prioritised time for workgroup meetings and displayed high levels of accountability in mobilising their teachers. Food and Hospitality’s leader explained her efforts in between workgroup meetings as: “scheduling interim sessions to keep everyone up-to-date and prepare [for the upcoming workgroup sessions], I have to keep them [the teachers] on their toes, but that is my role.” As part of the workgroup meetings in the project’s first year, participants reflected on the way citizenship education was currently provided, its pros and cons, what they would like to change, and how they could include students in the process of improving citizenship education. Both programmes would quickly come up with ideas for involving students and both also had someone, other than the educational leader, who would be in charge. However, these ideas would often centre around simply engaging in conversation with students, which turned out to be quite challenging. Most VET students are seldom asked for their perspectives and therefore are unlikely to share much on a first try. Rather than being patient and persevering in order to spawn constructive participation, some staff were discouraged, saw their already low expectations being confirmed, and moved on to other tasks. Flawed implementation of student voice activities prevented these study programmes from accurately identifying student-raised issues as well as forging meaningful student-staff partnerships to develop potential solutions. Although the educational leaders from Hairdressing and Home and Personal Care Assistance started with similar enthusiasm and intensity, they were confronted with extensive differences in needs, competences, and backgrounds among the teachers in their programmes. Although both workgroups would come up with tasks to work on in between meetings, the majority failed to honour those agreements. Both programmes dealt with these complications in different ways. The disposition of the educational leader of Home and Personal Care Assistance shifted from being motivated and propagating a high level of involvement to a more wait-and-see attitude accompanied by lowering ambitions and eventually questioning the project’s relevance. Hairdressing’s management started behaving increasingly authoritative. Rather than cancelling or rescheduling certain meetings, they were adamant about the teachers’ presence and refused to be brushed aside, but nevertheless were unsuccessful in their endeavours. Moving from planning to impactful execution was further complicated by high degrees of administrative turnover during the project, which applied to every study programme. Frequent changes in leadership and participants resulted in complications in upholding strong communication and negotiation between stakeholders, reduced leadership familiarity with the project, altered institutional goals and priorities, and a lack of consistent advocacy for the project. Likewise, even though teachers who had implemented the curriculum negotiation tool were pleasantly surprised with and hopeful about the first results, educational leaders had difficulties with translating these results to the wider study programme as well as continuing the process of improving both citizenship education and the curriculum negotiation tool without the constant presence of EG.

### Theme 4: Tensions and intentions about roles and responsibilities between participants

Compared to more traditional interventions where stakeholders may be less involved, our participatory action project required high levels of active collaboration. Although the nature of the collaboration was discussed with and agreed to by every study programme, many teachers had different expectations about EG’s role: “In the beginning, I was doubtful about Esther [EG]. I thought she was the one who would show us the light […] Her role was not entirely clear to me […] At the previous meeting we agreed to contact other teachers (yes, that was my idea), but wasn’t the project or Esther [EG] supposed to do that?” (teacher, Hairdressing). The Hairdressing teachers were very opinionated about citizenship education and wanted to change many of its elements. However, after learning that this meant investing some of their own time and energy, they became very reluctant and would rather settle for leaving the subject as it was. Another example concerning communication and expectations occurred during a workgroup meeting with Food and Hospitality. At the request of the educational leader, a teacher had invited a student to join the gathering. While we were waiting for the student to arrive, it turned out that neither the teachers nor the educational leader had prepared anything. Instead, they urged EG to ask whatever she wanted to know since “it was you [EG] who wanted a student to participate” (educational leader, Food and Hospitality). Similar to the previous example, staff had come up with this specific plan of action themselves, rather than EG. Throughout the project, participants from various study programmes delegated responsibility and ownership in involving their students to EG: “You can just come to my class […] You are welcome to stop by and discuss something with my class” (teacher, Hairdressing). Divergent views on roles and responsibilities were also apparent between managers and teachers and among teachers. Citizenship teachers from Sports and Recreation and Food and Hospitality already worked together prior to the project, whereas the teachers from Hairdressing and Home and Personal Care Assistance had limited contact and hardly collaborated prior to the merge. Moreover, when setting up a workgroup, the educational leader of Hairdressing did not take personal interests or opinions into account, as she assumed the teachers did not want “another new thing”, but rather combined current citizenship teachers with those who might be teaching citizenship education the following year. Planning, dividing and executing tasks together with unfamiliar colleagues became even more difficult as many participants rejected their assigned roles and responsibilities, which in turn, created a vicious circle in which fewer and fewer participants actively collaborated.

## Discussion and conclusion

This study emphasises the promise of involving students and staff in teaching and learning in VET, providing four cases which illustrate various opportunities and challenges for education development in complex systems. The first research question focused on how to involve students and staff in co-creation and decision-making regarding citizenship education development. We established that the collaboration between students and staff particularly flourished while using the curriculum negotiation tool. The second research question concentrated on relevant factors influencing the process of involving students and staff, which were: alignment between project and participants’ priorities and goals, receptivity to student and teacher voices, leadership engagement, and tensions and intentions about roles and responsibilities between participants. We want to emphasise that the identified themes should not function as a checklist to be ticked off. Rather, attention should be paid to these themes throughout the project, underlining the continuous and sometimes unpredictable process of collaborative work in complex systems, in which stakeholders, priorities, contexts, and behaviours are constantly in motion and are repeatedly subject to change.

Initially, most participating study programmes in our project did not display environments receptive to student voices. This makes sense given that previous research in various educational settings has highlighted the prevailing dominance of behavioural control and teacher-led inquiry in classrooms, limiting opportunities for young people to practise autonomy (Ozer et al., 2010). This raises concerns about whether classrooms in its current form are fruitful environments for young people to flourish as learners, thinkers, and citizens of democratic societies (Kozol, 2012; Weinstein, 2002). By contrast, in the Netherlands, the overall trend in education has been shifting towards an environment reflective of inclusion, active participation, involvement in the learning process, and personal development, which is also reflected in the duty of VET institutions to provide citizenship education. Nevertheless, previous research established that traditional and teacher-centred methods still dominate Dutch citizenship education classrooms in VET (ECBO, 2019). Moreover, their study revealed that these students did not feel involved, nor did they believe that they were taken seriously by their teachers, echoing some of the past experiences of the students who participated in our study. These findings suggest that, similar to the views of some staff in our study, student voice opportunities are only available and desired for certain young people and not for others. PAR is “clearly counter-cultural insofar as it is fundamentally about student-led inquiry, the valuing of students’ concerns and expertise, and the opening up of opportunities for students to take on tasks and roles that involve self-regulation as well as participation in governance” (Ozer et al., 2010, p. 153). In other words, PAR has the potential of starkly disrupting the existing status quo (Ozer et al., 2008) and breaking through ingrained patterns and behaviours within complex systems to ensure influence for students from all levels of education. Gross (2004) established four levels of disturbance in order to describe the intensity of so-called ‘turbulence’: light (i.e., little to no disturbance); moderate (i.e., regular or buffeting disturbance, but sufficient stability to carry on); severe (i.e., increased disturbance leading to loss of stability, at least for some time); and extreme (i.e., salient disturbance resulting in structural damage). In our case, we observed that although the board of directors had marked promoting student voice as a priority, turbulence was too low at the classroom level, which is the chief setting where encounters between teachers and students occur. Hence, during the first two years, turbulence was increased to a moderate level by instructing staff to collect student perspectives in the classroom. Yet moving from planning to execution turned out to be very challenging and therefore, during the final year of the project, turbulence was raised even more by allowing students to design, develop and deliver citizenship education lessons. As expected, significant changes in roles and responsibilities of students and staff concerning citizenship education development initially led to considerable chaos and instability (Geurts et al., 2023). This resonates with the notion of complex adaptive systems which perceive tensions as natural phenomena. Such tensions are believed to stem from the desire to preserve the status quo and by extension the traditional division of tasks. Over time, existing relationships are redefined and new processes are developed and practised, which subsequently turn into professional patterns. To facilitate this process, roles and responsibilities need to be explicitly clarified and agreements about cooperation should be discussed in a transparent manner between and within groups of stakeholders (Huber, 2011). In our study, tense feelings only lasted for a short period of time, which is why we feel that just the right amount of turbulence was created in order to spark change and take a step in the right direction from narrow traditional cultural standards towards more inclusive and collaborative spaces in school environments.

### Strengths and limitations

To our knowledge, this is the first study focusing on involving VET students and staff in intervention development concerning teaching and learning. We included complete classes rather than self-selected students with the goal of making student voice opportunities accessible to every student. Accordingly, our notion that student voice activities should not be limited to high-achieving students, but should include students from all levels of education, also VET level 2 students, was confirmed. Conducting PAR via citizenship education proved to be an excellent fit and, especially in the final year, enabled us to spend most of the time in class committed to the implementation, reflection and improvement of the curriculum negotiation tool.

Some limitations are worth mentioning. First, due to the covid-19 pandemic, we encountered many interruptions during the first two years with online education and limited opportunities for interaction with students. For us researchers, it was not possible to directly contact students, which is why already early on teachers were left to their own devices in engaging students and delivering student voice activities. Without the pandemic, we could have positioned students more meaningfully in the role of innovators of the citizenship education curriculum. The pandemic and its aftermath primarily complicated the PAR process, rather than its content. For example, opportunities for face-to-face contact and building rapport and trust could have been an important facilitating factor that we did not have. That is why we suspect that our findings are an underestimation of the potential value and impact of promoting student voice in complex systems such as VET institutions. It should be encouraging that even under such pressing conditions, we were able to work collaboratively with teachers and students and construct a student-led curriculum. Second, study programmes were selected based on expressed interest, which could have led to somewhat one-sided results. Nevertheless, PAR assumes a certain degree of stakeholder interest and motivation from its participants. Moreover, we wanted to start with a few catalysts who would spread their knowledge, skills and objectives from the bottom up to their colleagues and other study programmes. Including programmes who showed an initial interest turned out to be a wise decision as staying motivated and engaged was challenging at various stakeholder levels throughout the project. Third, the participating study programmes primarily catered to students from service providing professions, which, in line with the previous limitation, has potentially complicated representation of the sample. Nonetheless, we have tried to capture a wide sample of participants which does justice to the immense size of the institution as well as the considerable diversity in teachers and students. As a result, we identified and addressed some core challenges of what complex systems have to deal with on a daily basis.

### Implications for practice and future research

In terms of implications for practice, we suggest to continuously attend to the identified themes, rather than assuming they have been sufficiently discussed and therefore can be ticked off. This is especially true for stakeholders’ understanding of student voice along with what degree of student voice is desired and with which students. Also, be transparent about what is minimally expected from each stakeholder in terms of delivering student voice activities. In our study, there was a clear mismatch between the overall vision of institutional policy makers favouring student involvement on the one hand and current practice as well as typical classroom relationships on the other hand. Yet, we also established that from the moment that teachers actually started working collaboratively with students, many of their doubts decreased, which, in turn, led to renewed motivation for becoming student voice advocates and planning new action steps. These findings underline the importance of quickly engaging staff in concrete initiatives. However, Mitra and Gross (2009) have pointed out that engaging in student voice activities comes with significant risks, particularly when such activities are poorly organised or are implemented inconsistently. Indeed, we confirmed that the implementation of many student voice activities during the first two years was flawed, which hampered appropriate identification of student-raised issues as well as complicated building rapport and trust between students and teachers. Thus, reaching consensus on shared goals and being involved in actually making something happen need to be carefully balanced and openly discussed. Furthermore, starting with a few motivated catalysts who spread their knowledge, skills and objectives bottom-up is good starting point, but should always be accompanied by facilitation, coordination, support and resources from middle management as well higher up in the organisation.

Future research should explore opportunities for more profound implementation of PAR. In our study, developing the intervention to improve citizenship education was mainly adult-led with students being consulted on numerous occasions. This resulted in the curriculum negotiation tool which allowed students to make all the decisions concerning the citizenship education curriculum, while being supported by their teachers. Additional research should seek ways of including students more thoroughly in intervention development as well as in scientifically studying the PAR process. Future endeavours should also focus on reaching a wider variety of study programmes and assess collaborations with stakeholders and institutions which may not show much interest at first.

## Conclusion

In conclusion, collaboration between students and staff particularly flourished while using the curriculum negotiation tool. Creating mission alignment and shared goals was complicated, because stakeholders had very diverse goals, which were not sufficiently connected. Even though many study programmes did not initially display an environment which valued student voice, three programmes implemented the curriculum negotiation tool, which resulted in improved expectations and more accurate impressions of their students. Moving from planning to suitable execution of student voice activities was challenging. Over time, staff took up more responsibility and ownership in terms of citizenship education development.

## References

[CR1] Askell-Williams, H. (2017). Perspectives from teachers and school leaders about long-term sustainability: A challenge for mental health promotion initiatives in educational settings. In C. Cefai & P. Cooper (Eds.), *Mental health promotion in schools* (pp. 141–155).

[CR2] Askell-Williams, H., & Murray-Harvey, R. (2016). Sustainable professional learning for early childhood educators: Lessons from an Australia-wide mental health promotion initiative. *Journal of Early Childhood Research*, *14*(2), 196–210.

[CR3] Berg, S., Bradford, B., Robinson, D. B., & Wells, M. (2018). Got health? Action researching a student-led health promotion program. *Canadian Journal of Action Research*, *19*(1), 33–47.

[CR4] Bergmark, U., & Westman, S. (2016). Co-creating curriculum in higher education: Promoting democratic values and a multidimensional view on learning. *International Journal for Academic Development*, *21*(1), 28–40.

[CR5] Bovill, C. (2014). An investigation of co-created curricula within higher education in the UK, Ireland and the USA. *Innovations in Education and Teaching International*, *51*(1), 15–25.

[CR6] Braun, V., & Clarke, V. (2006). Using thematic analysis in psychology. *Qualitative Research in Psychology*, *3*(2), 77–101.

[CR7] Brooker, R., & MacDonald, D. (1999). Did we hear you? Issues of student voice in a curriculum innovation. *Journal of Curriculum Studies*, *31*(1), 83–97.

[CR8] Cammarota, J., & Fine, M. (2010). Youth participatory action research: A pedagogy for transformational resistance. In J. Cammarota, & M. Fine (Eds.), *Revolutionizing education* (pp. 9–20). Routledge.

[CR9] Caraballo, L., Lozenski, B. D., Lyiscott, J. J., & Morrell, E. (2017). YPAR and critical epistemologies: Rethinking education research. *Review of Research in Education*, *41*(1), 311–336.

[CR10] Carey, P. (2013). Student as co-producer in a arketized higher education system: A case study of students’ experience of participation in curriculum design. *Innovations in Education and Teaching International*, *50*(3), 250–260.

[CR11] Coll, L., O’Sullivan, M., & Enright, E. (2018). The trouble with normal: (Re)imagining sexuality education with young people. *Sex Education*, *18*(2), 157–171.

[CR12] Cook-Sather, A. (2002). Authorizing students’ perspectives: Toward trust, dialogue, and change in education. *Educational Researcher*, *31*(4), 3–14.

[CR13] Cook-Sather, A. (2006). Sound, presence, and power: ‘Student voice’ in educational research and reform. *Curriculum Inquiry*, *36*(4), 359–390.

[CR14] Damschroder, L. J., Reardon, C. M., Widerquist, M. A. O., & Lowery, J. (2022). The updated Consolidated Framework for Implementation Research based on user feedback. *Implementation Science*, *17*(1), 75. 36309746 10.1186/s13012-022-01245-0 PMC9617234

[CR15] Dutch Expert Centre for Vocational Education and Training [ECBO]. (2019). *Eindrapportage MBO BurgerschapLabs [Final report VET CitizenshipLabs]*. CC BY.

[CR16] Dyson, B. P. (1995). Students’ voices in two alternative elementary physical education programs. *Journal of Teaching in Physical Education*, *14*(4), 394–407.

[CR17] Enright, E., & O’Sullivan, M. (2010). Can I do it in my pyjamas? Negotiating a physical education curriculum with teenage girls. *European Physical Education Review*, *16*(3), 203–222.

[CR18] Enright, E., & O’Sullivan, M. (2012). Producing different knowledge and producing knowledge differently: Rethinking physical education research and practice through participatory visual methods. *Sport Education and Society*, *17*(1), 35–55.

[CR19] EP-Nuffic. (2015). *Education system the Netherlands: The Dutch education system described*. EP-Nuffic.

[CR20] Fielding, M. (2001). Students as radical agents of change. *Journal of Educational Change*, *2*(2), 123–141.

[CR21] Fine, M., Torre, M. E., Burns, A., & Payne, Y. A. (2007). Youth research/participatory methods for reform. In D. Thiessen, & A. Cook-Sather (Eds.), *International handbook of student experience in elementary and secondary school* (pp. 805–828). Springer.

[CR22] Gosin, M., Dustman, P., Drapeau, A., & Harthun, M. (2003). Participatory action research: Creating an effective prevention curriculum for adolescents in the Southwestern US. *Health Education Research*, *18*(3), 363–379. 12828237 10.1093/her/cyf026

[CR23] Gregg, J., Solotaroff, R., Amann, T., Michael, Y., & Bowen, J. (2008). Health and disease in context: A community-based social medicine curriculum. *Academic Medicine*, *83*(1), 14–19. 18162745 10.1097/ACM.0b013e31815c67f0

[CR24] Gross, S. J. (2004). *Promises kept: Sustaining school and district leadership in a turbulent era*. ASCD.

[CR25] Guadalupe, T., & Curtner-Smith, M. D. (2020). It’s nice to have choices: Influence of purposefully negotiating the curriculum on the students in one mixed-gender middle school class and their teacher. *Sport Education and Society*, *25*(8), 904–916.

[CR101] Geurts, E. M. A., Reijs, R. P., Leenders, H. H. M., Simons, E. M. C., Jansen, M. W. J., & Hoebe, C. J. P. A. (2023). ‘I have discovered how to have faith in my students’: Negotiating a citizenship education curriculum with vocational education students. *Citizenship, Social and Economics Education*, *22*(3), 189–203.

[CR100] Geurts, E. M. A., Reijs, R. P., Leenders, H. H. M., Jansen, M. W. J., & Hoebe, C. J. P. A. (2024). Co-creation and decision-making with students about teaching and learning: A systematic literature review. *Journal of Educational Change*, *25*(1), 103–125.

[CR26] Hargreaves, A., & Fink, D. (2012). *Sustainable leadership*. John Wiley & Sons, Inc.

[CR27] Howley, D., & Tannehill, D. (2014). Crazy ideas: Student involvement in negotiating and implementing the physical education curriculum in the Irish senior cycle. *Physical Educator*, *71*(3), 391–416.

[CR28] Huber, S. G. (2011). School governance in Switzerland: Tensions between new roles and old traditions. *Educational Management Administration & Leadership*, *39*(4), 469–485.

[CR29] Izumi, B. T., Peden, A. M., Hallman, J. A., Barberis, D., Stott, B., Nimz, S., Ries, W. R., & Capello, A. (2013). A community-based participatory research approach to developing the Harvest for Healthy Kids curriculum. *Progress in Community Health Partnerships: Research Education and Action*, *7*(4), 379–384. 24375178 10.1353/cpr.2013.0047

[CR30] Koh, G. A., & Askell-Williams, H. (2021). Sustainable school‐improvement in complex adaptive systems: A scoping review. *Review of Education*, *9*(1), 281–314.

[CR31] Kozol, J. (2012). *Savage inequalities: Children in America’s schools*. Crown.

[CR32] Lang, D. J., Wiek, A., Bergmann, M., Stauffacher, M., Martens, P., Moll, P., & Thomas, C. J. (2012). Transdisciplinary research in sustainability science: Practice, principles, and challenges. *Sustainability Science*, *7*, 25–43.

[CR33] Mason, M. (2008). What is complexity theory and what are its implications for educational change? *Educational Philosophy and Theory*, *40*(1), 35–49.

[CR34] McQuillan, P. J. (2008). Small-school reform through the lens of complexity theory: It’s good to think with. *Teachers College Record*, *110*(9), 1772–1801.

[CR35] Mitra, D. L., & Gross, S. J. (2009). Increasing student voice in high school reform: Building partnerships, improving outcomes. *Educational Management Administration & Leadership*, *37*(4), 522–543.

[CR36] Moore, G. F., Evans, R. E., Hawkins, J., Littlecott, H., Melendez-Torres, G., Bonell, C., & Murphy, S. (2019). From complex social interventions to interventions in complex social systems: Future directions and unresolved questions for intervention development and evaluation. *Evaluation*, *25*(1), 23–45. 30705608 10.1177/1356389018803219 PMC6330692

[CR37] Müller-Kuhn, D., Zala-Mezö, E., Häbig, J., Strauss, N., & Herzig, P. (2021). Five contexts and three characteristics of student participation and student voice: A literature review. *International Journal of Student Voice*, *6*(2).

[CR38] O’Mara-Eves, A., Brunton, G., Oliver, S., Kavanagh, J., Jamal, F., & Thomas, J. (2015). The effectiveness of community engagement in public health interventions for disadvantaged groups: A meta-analysis. *Bmc Public Health*, *15*, 1–23. 25885588 10.1186/s12889-015-1352-y PMC4374501

[CR39] Oliver, K. L., & Kirk, D. (2015). *Girls, gender and physical education: An activist approach*. Routledge.

[CR40] Ozer, E. J., Cantor, J. P., Cruz, G. W., Fox, B., Hubbard, E., & Moret, L. (2008). The diffusion of youth-led participatory research in urban schools: The role of the prevention support system in implementation and sustainability. *American Journal of Community Psychology*, *41*, 278–289. 18299977 10.1007/s10464-008-9173-0

[CR41] Ozer, E. J., Ritterman, M. L., & Wanis, M. G. (2010). Participatory action research (PAR) in middle school: Opportunities, constraints, and key processes. *American Journal of Community Psychology*, *46*, 152–166. 20676754 10.1007/s10464-010-9335-8 PMC2921496

[CR42] Pennisi, A. C. (2013). Negotiating to engagement: Creating an art curriculum with eighth-graders. *Studies in Art Education*, *54*(2), 127–140.

[CR43] Plsek, P. E., & Greenhalgh, T. (2001). The challenge of complexity in health care. *Bmj*, *323*(7313), 625–628. 11557716 10.1136/bmj.323.7313.625 PMC1121189

[CR44] Regeer, B. J., & Bunders-Aelen, J. G. (2009). *Knowledge co-creation: Interaction between science and society. A Transdisciplinary Approach to Complex Societal Issues*. Advisory Council for Research on Spatial Planning, Nature and the Environment/Consultative Committee of Sector Councils in the Netherlands [RMNO/COS].

[CR45] Rodríguez, L. F., & Brown, T. M. (2009). From voice to agency: Guiding principles for participatory action research with youth. *New Directions for Youth Development*, *2009*(123), 19–34. 10.1002/yd.312 19830799

[CR46] Shilcutt, J. B., Oliver, K. L., & Aranda, R. (2021). You want to get us involved more: Authorizing student voice in a dance setting. *Journal of Dance Education*, *23*(2), 1–11.

[CR47] Shilcutt, J. B., Oliver, K. L., & Aranda, R. (2022). I wish dance class NEVER ended: An activist approach to teaching dance. *Journal of Dance Education*, *22*(2), 108–118.

[CR48] Signal, L. N., Walton, M. D., Mhurchu, N., Maddison, C., Bowers, R., Carter, S. G., Gorton, K. N., Heta, D., Lanumata, C., T. S., & McKerchar, C. W. (2013). Tackling ‘wicked’ health promotion problems: A New Zealand case study. *Health Promotion International*, *28*(1), 84–94. 22419621 10.1093/heapro/das006

[CR49] Trombly, C. E. (2014). Schools and complexity. *Complicity: An International Journal of Complexity and Education*, *11*(1), 40–57.

[CR50] Wahl-Alexander, Z., Curtner-Smith, M., & Sinelnikov, O. (2016). Influence of a purposefully negotiated season of sport education on one teacher and his pupils. *European Physical Education Review*, *22*(4), 450–464.

[CR51] Waterlander, W. E., Pinzon, L., Verhoeff, A., Den Hertog, A., Altenburg, K., Dijkstra, T., Halberstadt, C., Hermans, J., Renders, R., C., & Seidell, J. (2020). A system dynamics and participatory action research approach to promote healthy living and a healthy weight among 10-14-year-old adolescents in Amsterdam: The LIKE programme. *International Journal of Environmental Research and Public Health*, *17*(14), 4928. 32650571 10.3390/ijerph17144928 PMC7400640

[CR52] Weinstein, R. S. (2002). Overcoming inequality in schooling: A call to action for community psychology. *American Journal of Community Psychology*, *30*(1), 21–42. 11928775 10.1023/A:1014311816571

